# Formation of Thermally Stable, High-Areal-Density, and Small-Diameter Catalyst Nanoparticles via Intermittent Sputtering Deposition for the High-Density Growth of Carbon Nanotubes

**DOI:** 10.3390/nano12030365

**Published:** 2022-01-24

**Authors:** Hirofumi Koji, Yuji Kusumoto, Akimitsu Hatta, Hiroshi Furuta

**Affiliations:** 1 School of Systems Engineering, Kochi University of Technology, 185 Miyanokuchi, Kami, Kochi 782-8502, Japan; kusumoto.yuji@gmail.com (Y.K.); hatta.akimitsu@kochi-tech.ac.jp (A.H.); 2 National Institute of Technology, Kitakyushu College, 5-20-1 Shii, Kokuraminami-ku, Kitakyushu, Fukuoka 802-0985, Japan; 3 Center for Nanotechnology, Research Institute, Kochi University of Technology, 185 Miyanokuchi, Kami, Kochi 782-8502, Japan

**Keywords:** carbon nanotubes, carbon nanotube forest, thermal CVD, catalyst particles, magnetron sputtering deposition, intermittent sputtering, oxidation, agglomeration, annealing, electric conductance, XRR

## Abstract

We report the formation of thermally stable catalyst nanoparticles via intermittent sputtering deposition to prevent the agglomeration of the nanoparticles during thermal chemical vapor deposition (CVD) and for the high-density growth of carbon nanotubes (CNTs). The preparation of high-areal-density and small-diameter catalyst nanoparticles on substrates for the high-density growth of CNTs is still a challenging issue because surface diffusion and Ostwald ripening of the nanoparticles induce agglomeration, which results in the low-density growth of large-diameter CNTs during high-temperature thermal CVD. Enhancing the adhesion of nanoparticles or suppressing their diffusion on the substrate to retain a small particle diameter is desirable for the preparation of thermally stable, high-areal-density, and small-diameter catalyst nanoparticles. The intermittent sputtering method was employed to deposit Ni and Fe metal nanoparticles on a substrate for the synthesis of high-areal-density CNTs for Fe nanoparticle catalyst films. The metal particles deposited via intermittent sputtering with an interval time of over 30 s maintained their areal densities and diameters during the thermal CVD process in a vacuum for CNT synthesis. An interval of over 30 s was expected to oxidize the metal particles, which resulted in thermal stability during the CVD process. The intermittent sputtering method is thus a candidate process for the preparation of thermally stable catalyst films for the growth of a high density of long CNTs, which can be combined with the present CNT production process.

## 1. Introduction

The physical properties of carbon nanotubes (CNTs), including their optical properties, mechanical stiffness, electrical conductivity, and thermal conductivity, are known to be derived directly from their structural features of chirality, diameter, length, layer numbers, defects, and areal density [1,2,3]. The chirality of single-walled CNTs (SWNTs) can be successfully controlled by pretreatment of the catalysts [4], such as Co-W bi-metal catalyst [5], and cloning of nanocarbon segments using organic synthesis, which has been reported in review articles [6,7]. Color variations of SWNTs have also been successfully controlled using chiral selective SWNTs [8,9].

The optical properties and thermal conductivity of CNT forests are highly dependent on the areal density of the CNT forest. However, process development for the areal density and diameter controls of multi-walled CNTs (MWNTs) remain challenges for the development of CNTs for applications.

High-areal-density growth and diameter controls of CNTs have been developed for a wide range of applications [10]. It is essential to study the formation of nanosized catalyst particles for the control of CNT growth. The formation of catalyst nanoparticles is explained by the dewetting of catalysts films into nanoparticles at high temperatures [11] observed using environmental TEM [12,13], and by Ostwald ripening of catalyst particles at high temperatures in vacuum [14,15]. An increase in the areal density of CNTs was reported following the plasma pretreatment of Fe catalysts for CNT growth, which resulted in an areal density of 4.8 × 10^12^ CNTs/cm^2^ [16], and the plasma treatment of an AlOx supporting layer before catalyst deposition resulted in an areal density of 1.5 × 10^13^ CNTs/cm^2^ [17]. To characterize these catalyst nanoparticles and metal catalyst layers in detail, high-resolution microscopy techniques such as scanning electron microscopy (SEM) [18,19,20], atomic force microscopy (AFM) [21,22,23], and transmission electron microscopy (TEM) [19,24] are utilized. These techniques are powerful tools for the direct observation of nanostructures and nanoparticles to study the behavior of catalysts during the preparation process, catalyst particle formation, and even during chemical vapor deposition (CVD). 

We have reported high-density CNT growth on a multi-layered Ni/Fe catalyst [25,26], in which a thin Ni (0.8–1.6 nm thick) over-coating layer could prevent the agglomeration of iron particles at high temperatures before the introduction of the carbon gas source. CNTs with an areal density of 3.5 × 10^11^ CNTs/cm^2^ were successfully grown on these substrates with a 1.0 nm thick Ni over-coating layer [25]. However, there are issues with these catalyst films with regard to large-scale deposition because the precise control of the thin Ni over-coat layer thickness (ca. 0.8 nm) is difficult for a large sample area. 

There have been intensive studies on bi-metal catalyst nanoparticles, such as a Co-Mo catalyst [27], MgO supported FeCu catalyst [28], CoCu [29], and FeMo [30], which can produce small-diameter carbon nanotubes, including chirality controlled SWCNTs. However, it is still considered challenging to develop a method for the uniform formation of bi-metal catalysts over a large area via sputtering deposition. The sputtering deposition of single-layer mono-metallic catalysts is expected to have the ability to control film thickness for large-scale deposition, compared with the thickness control of bi-layer or bi-metal catalyst films. In this paper, we investigate the control of size and areal density of mono-metallic catalysts using the intermittent sputtering method for the synthesis of high-density and small-diameter carbon nanotubes.

In situ observation of such thin films is still a challenge when attempting to achieve precise thickness control at the nanoscale. To characterize these metal catalyst films, we have previously proposed an electrical characterization method (intermittent sputtering) for films with a sub-nanoscale thickness, which involves the measurement of electrical conductivity during the interval between magnetron sputtering and the analysis of island formation during sputtering deposition. We evaluated the quality of monometallic Ni [31,32] and Fe metal catalyst films using this method, followed by CNT growth on a Fe catalyst. Fe catalysts on an AlO buffer layer on Si substrates (Fe/AlO/Si) are commonly utilized as CNT catalysts, on which high-areal-density and small-diameter CNTs are obtained. To evaluate the advantage of the intermittent sputtering deposition of Fe catalyst, compared with the conventional sputtering method, we used an Fe catalyst with AlO/Si substrate for the CNT synthesis.

## 2. Experimental Section

The surface morphology, smaller particle-like or granular nanoisland structures on the substrate, was observed after the deposition of a Ni and Fe metal via direct current (DC) magnetron intermittent sputtering (apparatus [31]) on thermally oxidized silicon (th-SiO/Si). In this paper, we use “nanoparticles” to mean these particle-like or granular nanoisland structures. A CNT forest was grown using thermal CVD after the deposition of Fe metal catalysts via DC magnetron intermittent sputtering on AlOx/Si substrates. Table 1 shows the conditions of each catalyst on substrate.

The AlOx layer was deposited on Si substrates via RF magnetron sputtering using an Al_2_O_3_ target, and was 30 nm thick. The control parameters for the DC magnetron sputtering system were the ON time for plasma sputtering during one cycle, the OFF time without sputtering for one cycle, and the ON/OFF time ratio and cycle number. The ON/OFF and OFF/ON transition times were less than 20 ms. A 50 mm diameter and 0.1 mm thick Ni plate (3N purity) was used as the sputtering target and was set on the DC magnetron cathode (TM2, Kurt J. Lesker Company, Jefferson Hills, PA, USA). A 50 mm diameter and 0.5 mm thick Fe plate (4N purity) was used as the sputtering target in addition to the Ni plate. After setting the substrate, the vacuum chamber was evacuated using a turbo molecular pump until the base vacuum pressure reached 3.0 × 10^−3^ Pa. After evacuation, a pre-discharge was performed to remove the surface oxide layer from the target with a closed shutter on the sample stage and the following conditions: an Ar flow rate of 10 sccm, a pressure of 0.8 Pa, a pre-sputtering duration of 10 min, and a discharge current of 40 mA.

Intermittent sputtering deposition was performed with a total sputtering ON time of 55 s (2 nm thick Fe film) and arbitrary OFF times (e.g., 1 s ON time and 10 s OFF time have 55 intervals sputtering cycles). The deposited Ni layer thickness was ca. 2 nm with a total sputtering time of 40 s. All of the sputter depositions in this study were conducted without substrate heating. The substrate temperature did not increase, which was confirmed by the sample temperature after deposition. The deposition conditions were an Ar flow rate of 10 sccm, a sputtering pressure of 0.8 Pa, a DC discharge current of 20 mA, and a distance between the target and substrate of 76 mm.

**Figure 1:**
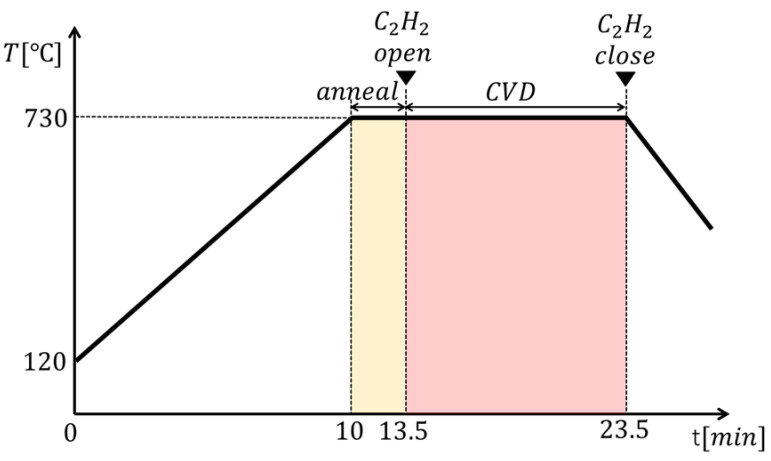
A temperature profile of thermal CVD for the growth of CNTs.

CNTs were grown via catalytic thermal CVD with acetylene (C_2_H_2_) as a source gas using Fe films of various thicknesses deposited on alumina (AlOx) buffer, layer-coated Si substrates. Figure 1 shows a temperature profile of the thermal CVD process for the growth of CNTs. The AlOx/Si substrates with the Fe film were kept in the CVD chamber at 120 °C, and the CVD chamber was evacuated to a base vacuum pressure of 5.0 × 10^−4^ Pa. The temperature of the CVD chamber was elevated from 120 °C to a growth temperature of 730 °C at a rate of 50 °C/min. After reaching 730 °C, the substrates were annealed in a vacuum for 3.5 min, and the C_2_H_2_ source was introduced into the chamber at a flow rate of 10 sccm. The CVD process was performed at a growth pressure of 54 Pa and a growth temperature of 730 °C for 10 min. 

The surface morphology of the as-deposited Ni and Fe catalysts on the th-SiO/Si substrates was observed using AFM. The annealing process for the catalyst on the substrates was performed in the vacuum chamber at 730 °C for 3.5 min under the same conditions as the CVD process without the C_2_H_2_ gas supply. The surface morphologies of the specimens after Ni deposition were investigated using field-emission scanning electron microscopy (FE-SEM) JSM-7401 (JEOL Ltd., Akishima, Tokyo, Japan), and after Fe deposition on the AlOx/Si substrate, were investigated using AFM. The Ni films deposited via intermittent sputtering were characterized using electrical conductance measurements. The electrical conductance of the Ni films was measured via the application of a voltage to the electrodes on the substrate surface during each OFF time. The surface condition of the deposited Ni films, including the surface roughness, thickness, and density, were evaluated by X-ray reflectance (XRR) measurements using a high-resolution X-ray diffractometer (Advanced Thin Film X-ray System ATX-G, Rigaku Co., Akishima, Tokyo, Japan).

## 3. Results and Discussion

### 3.1. Effect of ON/OFF Time Ratio of Intermittent Sputtering on Particle Size Distributions

**Figure 2:**
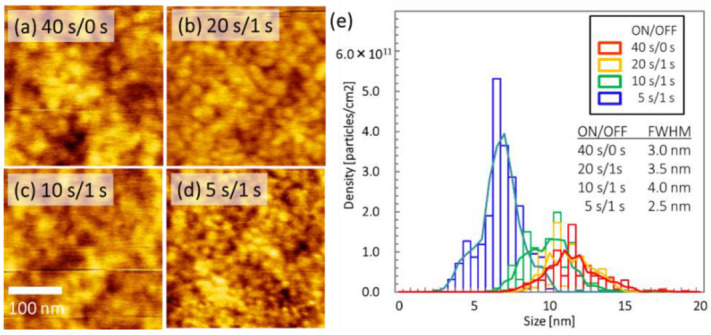
AFM images of as-deposited, 2 nm thick Ni films on th-SiO substrates deposited via (**a**) continuous DC sputtering and intermittent sputtering with ON times of (**b**) 20 [32], (**c**) 10, and (**d**) 5 s [32], and a constant OFF time of 1 s. (**e**) Distribution of Ni particle sizes. The particle size was reduced with decreasing ON/OFF time ratio.

Figure 2 shows AFM images of the surface morphology of Ni metal films deposited via intermittent sputtering on th-SiO/Si substrates. The ON/OFF time ratio was varied for intermittent sputtering of Ni, where the OFF time was 1 s and the ON time was 5, 10, or 20 s. The total ON time was 40 sec and the total OFF times were 7, 3, and 1 s, correspondingly.

The surface morphology had smaller nanoparticles on the substrate for an ON time of 5 s than for an ON time of 20 s, as shown in Figure 2a–d. The particle size distributions determined from these AFM images are plotted in Figure 2e. The areal densities of particles are shown in Table 2. A shorter ON time resulted in a higher areal density.

The FWHM of the particle size distributions is shown in Table 2. The particle density was almost the same for intermittent sputtering with an OFF time of 1 s as for continuous sputtering. These results demonstrate that intermittent sputtering with a lower ON/OFF ratio could deposit a high areal density of small particles on the substrate. The route mean squared (RMS) roughness (Sq) for these Ni films, calculated from the AFM images, was 0.246 nm (ON/OFF = 5 s/1 s), 0.211 nm (ON/OFF = 10 s/1 s), 0.207 nm (ON/OFF = 20 s/1 s), and 0.203 nm (ON/OFF = 40 s/0 s). The RMS value increased slightly with a decreasing ON/OFF time ratio. A possible interpretation is that larger particles were formed with higher mobility, which form flat surfaces on the substrates via the Frank-van der Merwe growth mode for a higher ON/OFF time ratio, whereas nanoparticles of smaller particles were formed with lower mobility, producing rough surfaces via the Volver–Weber (VW) growth mode for a lower ON/OFF time ratio. 

**Figure 3:**
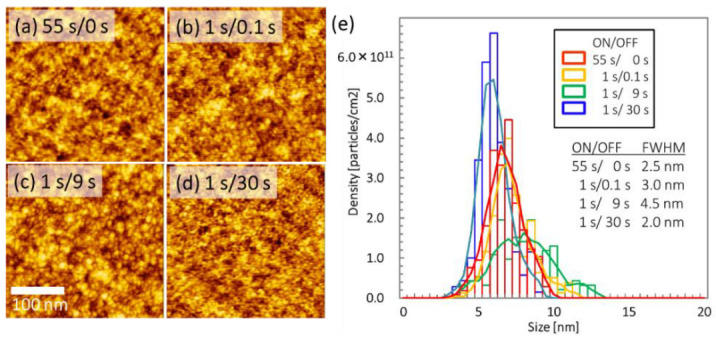
AFM images of as-deposited, 2 nm thick Fe films on th-SiO/Si substrates deposited via (**a**) continuous DC sputtering and by intermittent sputtering with ON times of (**b**) 0.1, (**c**) 9, and (**d**) 30 s, and a constant OFF time of 1 s. (**e**) Distribution of Fe particle sizes. The particle size was reduced with decreasing ON/OFF time ratio.

Figure 3 shows AFM images of the substrate surface morphology for Fe metal deposited via intermittent sputtering on th-SiO/Si substrates with various ON/OFF ratios. The ON time was constant at 1 s, and the OFF times were 0.1, 9, and 30 s for one cycle. The total OFF time was 5.4 s for ON times of 1 s and 1620 s for ON times of 1 s in Figure 2b,d. Smaller particles were observed for an OFF time of 30 s than for 0.1 s, as shown in Figure 3a–d.

The particle size distributions obtained from these AFM images are plotted in Figure 3e. The areal densities of particles on the substrate surface and the FWHM are shown in Table 3. The areal density is non-linear with respect to the OFF time, and the longest OFF time of 30 s gave the highest areal density of 2.7×10^12^ /cm^2^. The FWHM for the particle size distributions was 2.0 nm, which is the approximate FWHM for a 30 s OFF time. These results indicate that a smaller ON/OFF ratio, i.e., a longer OFF time of 30 s during one cycle, can deposit a high areal density of small particles on the substrate.

### 3.2. Effect of Catalyst Aggregation Prevention during an Annealing Process

**Figure 4:**
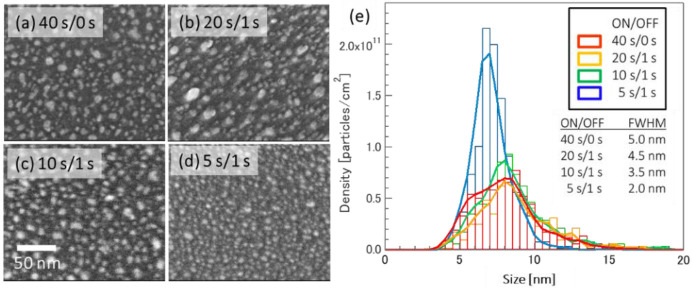
SEM images of annealed, 2 nm thick Ni films on th-SiO/Si substrates deposited via (**a**) continuous DC sputtering, and by intermittent sputtering with ON times of (**b**) 20, (**c**) 10, and (**d**) 5 s with a constant OFF time of 1 s. (**e**) Distribution of Ni particle sizes after annealing. The particle size was reduced with decreasing ON/OFF time ratio.

Figure 4 shows FE-SEM images of the surface morphology of Ni metal films deposited via intermittent sputtering on th-SiO/Si substrates. The substrate was annealed at 730 °C for about 3.5 min, which simulates the surface condition just before the source gas is supplied in the CNT growth process. The reason for using FE-SEM for the morphology of Ni/th-SiO/Si substrate after annealing is that the substrate surface roughness is larger after annealing than before, so it is difficult to perform accurate observations using AFM. Note that it is not possible to simply compare the particle size and areal density due to the different measurement principles between FE-SEM and AFM. Figure 4a–d show FE-SEM images of the substrate surface morphology after annealing samples produced using different OFF times. The surface was covered by Ni metal particles, which were smaller for an ON time of 5 s than for longer ON times. This is consistent with the results for as-deposited particles. 

The particle size distribution is shown in Figure 4e. The areal densities of the particles on these annealed substrate surfaces and the FWHM are shown in Table 4. The areal density of 1.0 × 10^12^ /cm^2^, which is approximately two times higher, was achieved with a 5 s ON time. The FWHM of the particle size distributions was approximately half at 2.0 nm with a 5 s ON time. In addition, a lower ON/OFF time ratio prevented the agglomeration of the as-deposited particles at high annealing temperatures, while Ni particles deposited via continuous sputtering were agglomerated, which suggests the oxidation of the deposited Ni particles during the OFF time.

### 3.3. CNT Growth on Fe Catalyst Films Deposited via Intermittent Sputtering Method on AlOx/Si Substrates

**Figure 5:**
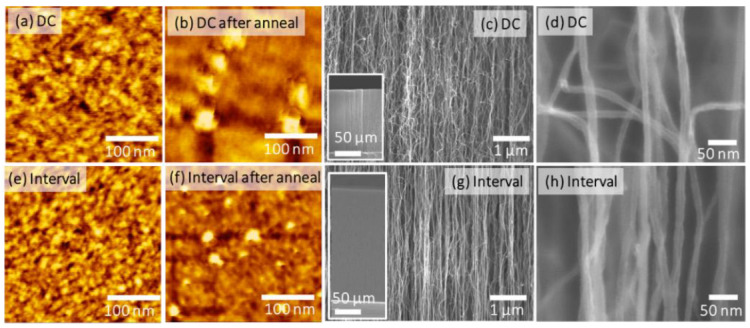
Surface AFM images of Fe catalyst films deposited on AlOx substrate via continuous sputtering; (**a**) as-deposited sample and (**b**) after the annealing process; intermittent sputtering with an OFF time of 30 s for the (**e**) as-deposited sample and (**f**) after the annealing process. Cross-sectional SEM images of (**c**,**d**) CNT forest grown on continuous sputtering catalyst and (**g**,**h**) CNT forest grown on intermittent sputtering catalyst.

Figure 5 shows AFM images of the surface morphologies of as-deposited and as-annealed Fe metal films deposited via DC and intermittent sputtering on AlOx/Si substrates, and FE-SEM images of a CNT forest grown via thermal CVD using the Fe metal catalysts. AFM images before and after annealing of the Fe metal film deposited via continuous DC sputtering are shown in Figure 5a,b, and those deposited via intermittent sputtering with a 30 s OFF time are shown in Figure 5e,f. The introduction of intermittent sputtering OFF time reduced the size of particles, which are considered to be composed of Fe, in as-deposited and annealed samples on AlOx/Si substrates, which is consistent with the results for Ni on th-SiO/Si substrates in Section 3.2. Similar to the case of the th-SiO/Si substrates, the AlOx/Si substrate also exhibited smaller particles following intermittent sputtering before and after annealing, as discussed in Section 3.1 and Section 3.2.

Cross-sectional SEM images of CNTs grown on the Fe catalysts are shown in Figure 4c,d for Fe catalysts deposited via DC sputtering, and in Figure 5g,h via intermittent sputtering. The agglomeration of Fe particles deposited via intermittent sputtering was suppressed during high-temperature annealing, whereas agglomeration was observed in the film produced via continuous sputtering, which suggests that the deposited Fe particles were oxidized during the OFF time for intermittent sputtering.

For continuous sputtering, when the AlOx/Si substrate was annealed, only large Fe particles (about 30 nm) were observed. However, for intermittent sputtering, when the substrate was annealed, many particles with sizes of 10 nm or less were observed, similar to the case for the unannealed substrate. These results show that the interval time has a more substantial effect on suppressing aggregation during high-temperature annealing for AlOx substrates than for SiO_2_ substrates.

Figure 5c shows cross-sectional SEM images of a CNT forest with a height of 202 μm, an areal density of 2.17 × 10^10^ CNTs/cm^2^, and an average CNT diameter of 13.0 nm on Fe catalysts deposited via intermittent sputtering. Figure 4g shows cross-sectional SEM images of the CNT forest with a height of 133 μm, an areal density of 1.85 × 10^10^ CNTs/cm^2^, and an average CNT diameter of 13.8 nm on the Fe catalysts deposited via continuous sputtering. CNT forests on Fe catalysts deposited via intermittent sputtering had longer, higher-areal density, smaller-diameter CNTs than those grown on Fe catalysts produced via continuous sputtering. 

In Figure 5d,h, the CNT forest is composed of straighter CNTs on the Fe catalyst deposited via intermittent sputtering compared with those deposited via continuous sputtering. This is due to the narrower size distribution of the catalyst particles deposited via intermittent sputtering, which resulted in a uniform growth rate of the CNTs to produce straight CNTs.

### 3.4. Electrical and X-ray Characterization of Metal Nanoparticles Deposited via Intermittent Sputtering Method 

**Figure 6:**
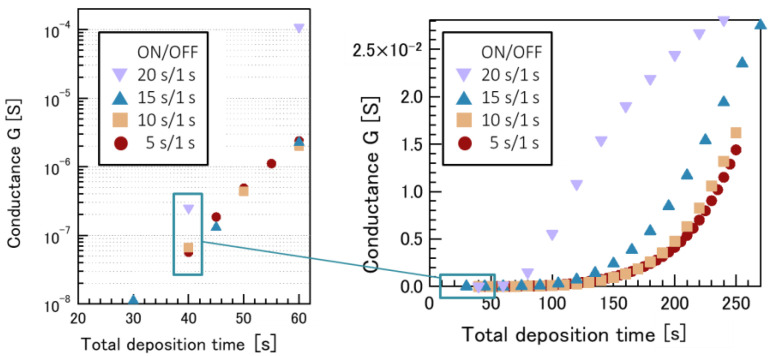
Time evolution of conductance G, for Ni catalyst films deposited with various ON/OFF time ratios via the intermittent sputtering method [32].

The Ni films deposited via intermittent sputtering were characterized using electrical conductance and XRR measurements to analyze the surface states of the films in detail. Figure 6 shows the variation of the conductance with the deposition time, where the deposition time is the total ON time, and the OFF time is not shown in the plot. The conductance increased with increasing deposition time. For the same total deposition time, the conductance was lower for a lower ON/OFF time ratio, i.e., for a shorter ON time.

We previously reported a decrease in conductance for Ni films deposited under a higher base pressure due to the formation of oxidized nickel films by the oxygen remaining in the vacuum chamber at higher oxygen partial pressures [31]. Figure 4 shows that for a lower ON/OFF time ratio, the higher areal density of grain boundaries and the smaller size of Ni nanoparticles causes a reduction in the conductance of the Ni films. 

**Figure 7:**
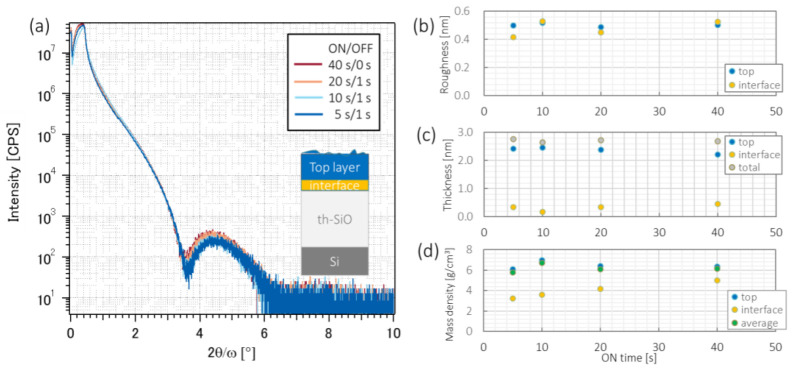
(**a**) XRR spectra of Ni films for various ON/OFF times in which the total deposition time was 40 s for each sample. (**b**) Roughness, (**c**) thickness, and (**d**) mass densities analyzed from XRR spectra for various ON/OFF time ratios with the same OFF time.

XRR measurements were conducted to analyze the surface properties of the Ni films in detail. Figure 7 shows the results of the XRR measurements for Ni films deposited via intermittent sputtering with various ON times and a constant total deposition time of 40 s. The variation in the roughness, thickness, and mass density and their dependence on the ON time are plotted in Figure 7b–d.

The surface roughnesses of the Ni films were 0.499 nm (ON/OFF = 5 s/1 s), 0.530 nm (ON/OFF = 10 s/1 s), 0.490 nm (ON/OFF = 20 s/1 s), and 0.502 nm (ON/OFF = 40 s/1 s). Based on the XRR spectra, the Ni films were composed of a top layer and an interface layer. A higher ON/OFF time ratio tended to decrease the thickness of the top layer and increase the thickness of the interface layer. Mass density generally decreases as a metal is oxidized, which suggests that the particles on the substrate surface were oxidized during the OFF time. The mass density of the top layer was lowest for the shortest ON time of 5 s; therefore, it is considered that the top layer was oxidized for a shorter ON time. 

The prevention of the agglomeration of the metal particles during annealing suggests that the particles were oxidized via intermittent sputtering for a lower ON/OFF time ratio. The melting point of metals (e.g., nickel) generally decreases when they form nanostructures for CNTs growth. Therefore, the particles of nanostructured metals for the growth of CNTs at high temperatures were agglomerated and the particles became large. On the other hand, the melting point of some oxidized metals can be higher than that for pure metals. (For example, the melting point of bulk Ni metal is about 1500 °C, and that of nickel oxide is about 2000 °C.) Furthermore, it has been reported that oxidized iron particles prevent agglomeration over 600 °C [33]. The particle-like structures deposited by intermittent sputtering were thus considered to avoid agglomeration and to a maintain high density under high-temperature CNT synthesis conditions. 

Oxidized metallic monolayer catalysts formed via interval sputtering are expected to provide more precise control of film thickness for large-scale deposition than bilayer or bimetallic catalysts. In addition, the intermittent sputtering method oxidizes metal particles on the substrate surface and can thus be combined with other film synthesis methods of producing high-areal-density and small-particle-size catalysts, such as bi-metal, bi-layered catalyst films. 

## 4. Conclusions

The effect of the ON/OFF time ratio for intermittent sputtering on the thermal stability of catalyst nanoparticles was examined to prevent the agglomeration of catalyst nanoparticles during the high-temperature thermal CVD process and thus achieve the high-density growth of CNTs. 

It was confirmed that the Ni and Fe catalysts deposited via intermittent sputtering with a lower ON/OFF time ratio (ON/OFF < 1 s/30 s) maintained their high areal density, small diameter, and narrow particle-size distribution at the thermal CVD temperature in a vacuum for CNT synthesis. 

To prepare Fe/AlOx multi-layer catalyst films, the surface morphologies of Fe catalyst particles on AlOx films were examined before and after the annealing process for the CNT synthesis by CVD. The small size and narrow FWHM for the Fe catalyst particles produced by intermittent sputtering with a lower ON/OFF time ratio contributes to the growth of CNTs with a small average diameter of 13 nm, a higher areal density (2.17 × 10^10^ CNTs/cm^2^) CNT forest, and thicker (202 μm) CNT forests.

A detailed analysis of the catalyst films was conducted using electrical resistance measurement and XRR analysis, which revealed that metal films deposited with a longer total OFF time have a higher electrical resistance and a lower mass density. 

It was concluded that the increase in the melting point via the oxidation of the deposited films during intermittent sputtering could reduce the mobility of catalyst atoms on the substrate surface and into the substrate during the high-temperature annealing process, which results in the thermal stability of the catalyst, with the growth of high-areal-density and thicker CNT forests. The intermittent sputtering method is thus a candidate for use in the preparation of thermally stable catalyst films for the formation of high-density, long-length CNTs and can be combined with the existing catalyst preparation process for the growth of CNTs for industrial application. Further studies are needed to confirm the applicability of the intermittent sputtering of a single catalyst to uniformly form catalysts and CNT growth over a large area.

## Figures and Tables

**Table 1:** Experimental condition of each catalyst on substrate.

Catalyst	Substrate	Observation
Ni (2 nm thick of 40 s ON time)	th-SiO/Si (100 nm thick of th-SiO)	AFM (as deposition) FE-SEM (after annealing) electrical conductance XRR measurement
Fe (2 nm thick of 55 s ON time)	th-SiO/Si (100 nm thick of th-SiO)	AFM (as deposition)
Fe (2 nm thick of 55 s ON time)	AlOx/Si (30 nm thick of AlOx)	AFM (deposited catalyst) FE-SEM (grown CNT)

**Table 2:** As-deposited Ni particle characteristics determined from AFM images.

ON/OFF Time Ratio	Areal Densities	FWHM
40 s/1 s (continuous)	7.4 × 10^11^ /cm^2^	3.0 nm
20 s/1 s	7.6 × 10^11^ /cm^2^	3.5 nm
10 s/1 s	1.0 × 10^12^ /cm^2^	4.0 nm
5 s/1 s	2.2 × 10^12^ /cm^2^	2.5 nm

**Table 3:** As-deposited Fe particle characteristics determined from AFM images.

ON/OFF Time Ratio	Areal Densities	FWHM
55 s/0 s (continuous)	2.2 × 10^12^ /cm^2^	2.5 nm
1 s/0.1 s	1.9 × 10^12^ /cm^2^	3.0 nm
1 s/9 s	1.6 × 10^12^ /cm^2^	4.5 nm
1 s/30 s	2.7 × 10^12^ /cm^2^	2.0 nm

**Table 4:** Annealed Ni particle characteristics determined from AFM images.

ON/OFF Time Ratio	Areal Densities	FWHM
40 s/1 s (continuous)	7.2 × 10^11^ /cm^2^	5.0 nm
20 s/1 s	6.1 × 10^11^ /cm^2^	4.5 nm
10 s/1 s	7.3 × 10^11^ /cm^2^	3.5 nm
5 s/1 s	1.0 × 10^12^ /cm^2^	2.0 nm

## Data Availability

The datasets generated during the current work are available from the corresponding author on reasonable request.
